# Covid-19 related factors to food security and dietary diversity among urban households in western Oromia, Ethiopia

**DOI:** 10.1016/j.heliyon.2023.e14476

**Published:** 2023-03-13

**Authors:** Tamiru Yazew, Agama Daba, Lelisa Hordofa, Girma Garedew, Abdi Negash, Gizachew Merga, Tasama Bakala

**Affiliations:** a Department of Public Health, College of Health Sciences, Salale University, Fitche, 245, Ethiopia; b Department of Food and Nutritional Sciences, Faculty of Agriculture, Wollega University, Nekemte, 395, Ethiopia; c Department of Natural Resource Management, Faculty of Resource Management and Economics, Wollega University, Nekemte, 395, Ethiopia; d Department of Medical Laboratory Sciences, College of Health Sciences, Salale University, Fitche, 245, Ethiopia; e Department of Horticulture, College of Agriculture and Natural Resources, Salale University, Fitche, 245, Ethiopia

**Keywords:** Associated factors, Covid-19, Dietary diversity, Ethiopia, Food security, Urban, AOR, Adjusted Odd Ratio, CI, Confidence Level, COR, Crude Odd Ratio, EU, European Union, HFIAS, Household Food Insecurity Access Scale, NPC, National Planning Commission, SPSS, Statistical Package for Social Sciences, UN, United Nations, USD, United states Dollar, USDA, United States Department of Agriculture

## Abstract

**Background:**

This study aims to assess factors associated with food security and dietary diversity among poor urban households of western Oromia, Ethiopia, after the outbreak of the Covid-19 pandemic.

**Method:**

A cross-sectional, community-based study was conducted in May to June 2021 with 361 poor urban households in the Horo Guduru Wollega zone, western Oromia, Ethiopia. A pre-tested structured questionnaire was used to collect primary data. Twenty-four hour reminder points were used to assess household dietary diversity, and household food security was assessed using the Household Food Insecurity Access Scale tool. Data were evaluated using the statistical software SPSS version 25.0.

**Results:**

This study showed a prevalence of food insecurity in households of 59.6%. The mean and standard deviation of household dietary diversity values were 4.19 ± 1.844. Family size (AOR = 8.5; 95% CI:3.295–21.92), monthly income (AOR = 3.52; 95% CI; 1.771–6.986), dietary diversity (AOR = 8.5; 95% CI; 3.92–18.59), knowledge (AOR = 3.0, 95% CI = 1.08–)8.347), attitude (AOR = 8.35, 95% CI:3.112–22.39) and practices against Covid-19 (AOR = 2.12; 95% CI:1.299–11.4) were factors significantly associated with food insecurity. Variables like educational status (AOR = 3.46; 95% CI:1.44–8.312), increased family size after the Covid-19 pandemic (AOR = 2.26; 95% CI:1.02–5.04), food security (AOR = 6.7; 95% CI:4.01–19.01), knowledge (AOR = 3.96; 95% CI:1.57–10.0), attitude (AOR = 3.9; 95% CI:1.75–8.82) and practices toward coronavirus (AOR = 2.23; 95% CI:2.18–23.95) were predictors significantly associated with dietary diversity.

**Conclusion:**

This study concluded that family size, monthly income, and dietary diversity were factors contributed to household food security. On the other hand, variables such as educational status, family size, and food security were highly relevant factors for dietary diversity after the outbreak of the Covid-19 pandemic. Knowledge, attitudes, and practices were also variables related to both household food security and dietary diversity. Therefore, immediate interventions such as nutrition-specific interventions can be suggested to address food insecurity and problems of inadequate food intake in poor urban households. In addition, governmental and non-governmental organizations should raise awareness and policies to support those at higher risk by developing affordable, sustainable and targeted social protection systems that ensure food security and adequate dietary intake at the household level.

## 1 Introduction

The outbreak and progression of the Covid-19 pandemic have brought with them a new set of challenges affecting the world in unprecedented ways in all aspects of human life, including public health and well-being [[1], [2], [3], [4]]. Almost 150 million people worldwide are expected to fall into extreme poverty and food insecurity [5]. As of 2020, approximately 55% of food-insecure households in the United States participated in federal food assistance programs [6]. More than 21.2 million people experienced severe food insecurity during the lean season in West and Central Africa [7].

Factors contributing to the high prevalence of food insecurity include movement restrictions, market closures, food market and distribution channel disruptions, reduced food production, and other socioeconomic crises [[8], [9], [10]]. Various preventive measures are being implemented by governments to combat Covid-19. However, this can introduce additional barriers to food systems, including marketing, logistics, and trade, potentially affecting food safety [11,12]. For instance, many people around the world have also lost their jobs as a result of the Covid-19 lockdown [4,8,13]. Studies conducted in India showed that the pandemic triggered a severe mobility crisis with migrant workers in many major cities wanting to return to their hometowns [14], and that marginalized people were affected by the Covid-19 pandemic [15]. The situation during the Covid-19 pandemic was complicated by the influence of transnational migrant workers, which put pressure on Romania's relations with the EU and a strict lockdown on the Roma community [16].

After the onset of the Covid-19 pandemic, factors significantly associated with household food insecurity were monthly income, age, living in a rented house, and number of family members [17]. Disruptions in global food supply chains, loss of income and livelihoods due to the global economic recession, and uneven food price developments were triggered by a complex set of factors following the onset of the Covid-19 pandemic [18]. For example, Afghanistan has experienced a sharp increase in food shortages following the outbreak of the Covid-19 pandemic due to its dependence on neighboring countries [19].

Evidence shows that the Covid-19 pandemic has increased food insecurity in Mexico [20], South Asia [21], the United States [22], and Ethiopia [23]. According to a study conducted in India, household food insecurity increased sharply from 21% to 80% during the Covid-19 situation in 2020 [24]. Another study conducted in China found that lockdown measures had a significant negative impact on household food security and socio-demographic characteristics remained the key factors in household food insecurity (50%) during Covid-19 [25]. In addition, a study conducted in Africa reported that more than 40% of households reduced their consumption of staple foods, legumes, animal-derived foods, other vitamin A-rich vegetables, and other fruits, and lowered dietary diversity scores [26]. The lockdown led to a significant drop in the percentage consumption of legumes, chicken, meat, and dairy in India [27]. Furthermore, a study conducted in Saudi Arabia shows that changes in eating habits (meal time and the number of daily meals) during lockdown were more common among participants with severe food insecurity [28]. In Ethiopia, the low consumption of dairy products in the capital fell significantly from 56% to 45% in 2022 [29].

During the Covid-19 pandemic, according to an Iranian study, educational level, monthly income, nutritional knowledge, and access to credit were significantly associated with household food security and dietary diversity score [30]. Above secondary education, monthly income, and refrigerator ownership were associated with higher household food security, while family size >5 was a potential determinant of lower household food security [31]. In addition, a study conducted in Bangladesh found that age and monthly income are significantly related to food insecurity [[32], [33], [34]]. A study conducted in Bangladesh, being rural, having no formal education, having a job, and having a low monthly income were potential predictors of lower household food security and dietary diversity [35].

In a pandemic, government aid is essential to support the most vulnerable households as they face health and economic challenges [[36], [37], [38]]. But government aid is only effective if it reaches vulnerable households promptly, as high rates of urbanization and globalization of trade and travel have contributed to the cross-border spread of the virus. Along with the Covid-19 pandemic, there is a global transitory food security and nutritional crisis [[39], [40], [41]]. This effect has a strong and complex relationship to pre-existing structural weaknesses in developing countries including Ethiopia. As a result, appropriate measures to mitigate the impact of the pandemic on household food security and dietary diversity assessment are urgently needed, especially for the most vulnerable groups who live in urban areas.

### 1.1 Research questions

This research study aims to answer the following main research questions.

RQ1 What is the prevalence of food insecurity among poor urban households after the outbreak of the Covid-19 pandemic?

RQ2 What is the prevalence of dietary diversity scores among poor urban households after the outbreak of the Covid-19 pandemic?

RQ3 What are the main factors related to food insecurity in poor urban households after the Covid-19 pandemic emerged in Ethiopia?

RQ4 What are the main predictors of dietary diversity among poor urban households after the Covid-19 pandemic emerged in Ethiopia?

To answer these research questions, this study therefore aims to identify factors associated with household food security and dietary diversity among poor urban communities in Horo Guduru Wollega zone, Oromia, Ethiopia after the outbreak of the Covid-19 pandemic.

### 1.2 Food security and the Covid-19 pandemic

Food is closely linked to human culture, and improving our understanding of the cultural dimension of food security is increasingly recognized as an essential part of the transition to a sustainably healthier diet [42]. According to a review conducted in Belgium, gender, family and decision-making power play a crucial role in interacting with culture and its impact on food security [43]. Therefore, a wide scope to improve food security policies through better consideration of culture is crucial to overcome food and nutrition security in the long term. Despite concerted efforts and various UN programs to combat hunger, only short-term local results have been achieved [44].

Research has shown that the interaction between Covid-19 and the decline in economic activity will lead to increased food insecurity within and between countries [45]. More than 1.8 million children in Ethiopia, Kenya and Somalia need urgent treatment for life-threatening severe acute malnutrition [46]. In 2022, Ethiopia’s food aid needs are expected to reach record levels between June and September [47]. The impact of Covid-19 is particularly severe for people at the bottom of the food insecurity distribution [45].

The Covid-19 pandemic has impacted the already fragile livelihoods and food security of many Ethiopians [48]. Currently, Ethiopia’s National Planning Commission (NPC) forecasts that the economy will shrink by 2.8%–3.8% due to the pandemic, exacerbating extreme poverty and food insecurity [49]. Food insecurity and low dietary diversity have also been negatively impacted, particularly among urban populations dependent on daily income and informal workers [50]. Another study conducted in Ethiopia during the Covid-19 pandemic also shows that about 6.8% and 7.18% of urban households suffered from severe food insecurity from the first to the sixth round [51].

There are a number of factors associated with food insecurity following the outbreak of the Covid-19 pandemic. Frustrated by Covid-19 and price inflation, divorced households, day laborers, government employees and residents of highland, hotland and lowland areas faced higher odds of not being resilient to household food insecurity [52]. The Covid-19 situation remains precarious as the highly transmissible delta variant has recently spread and to this day has varied impacts on different regions of the country [53]. USDA Economic Research Service (ERS) food security forecasts continue to show a sharp rise in the number of people food insecure around the world due to the Covid-19 pandemic [54].

The current Covid-19 pandemic is also likely to result in temporary food insecurity in vulnerable countries such as Africa, Latin America, Oceania and Asia [55]. It has been reported that the pandemic has had a dramatic impact on the food system, with direct and indirect consequences for human, plant and animal life and livelihoods [56]. In sub-Saharan Africa, it has been found that female-headed households, the poor and the less formally educated appear to be more food insecure during this global pandemic [57]. In Burkina Faso, the negative impact on food security can be explained by a combination of factors such as rising food prices, falling household incomes and remittances [58]. The pandemic has led to a significant increase in food insecurity, exacerbating and exposing the vulnerability of poor communities [[59], [60], [61]].

The most affected dimension of food security globally is accessibility, with reasonably solid evidence suggesting that both financial and physical access to food has been disrupted [62]. However, in sub-Saharan Africa an increase in Covid-19 levels negatively affects all 4 indicators of food security without exception [63]. Covid-19 is having a major impact on global food security as the virus itself and policy responses, particularly lockdowns and social distancing regulations, have triggered a massive recession and significant disruption in food value chains [64]. Covid-19 threatened food security even in some developed countries, while developing countries are hardest hit due to their high dependency on securing their food supplies [65].

**Null hypothesis 1 (H1):** Covid-19 pandemic has no impact on food insecurity among urban households.

**Null hypothesis 2 (H2):** Covid-19 pandemic has no impact on dietary diversity among poor urban households.

**Null hypothesis 3 (H3):** There are no factors associated with food insecurity among poor urban households after the outbreak of the Covid-19 pandemic.

**Null hypothesis 4 (H4):** There are no factors associated with dietary diversity among poor urban households after the outbreak of the Covid-19 pandemic.

## 2 Method

### 2.1 Study design and setting

A community based cross sectional study was conducted from May to June 2021 in selected towns in the Horro Guduru Wollega zone, western Oromia region of Ethiopia. This period is considered post pandemic in many regions of the world. Thus, the “worse” of the pandemic in 2020 and 2021 was different especially when comparing these results to other studies that were conducted in 2020 [37,38]. The zone is located at latitudes 9010 N and 9050 N and longitudes 36,000 E and 36,050 E. It has a total area of 8097 km^2^. The capital of the zone is Shambu, located 314 km west of Addis Ababa. The region has a total population of 1,570,040, of which 785,515 are males and 784,525 are females. 64,739 or 11.36% of the population are urban dwellers. In the Horo Guduru Wollega zone of western Ethiopia, about 51.3% of households were found to be food insecure following the onset of the Covid-19 pandemic [66].

### 2.2 Eligibility criteria

All urban households living in the selected towns for at least six months were included in the study, while those from the countryside, who were ill with Covid-19 during the survey, were excluded from the study area.

### 2.3 Source and study population

All urban poor households who were dependent on food aids from government and non-government organizations in the selected districts of the Horo Guduru Wollega zone were used as the source population. All urban poor households who were dependent on food aids from government and non-government organizations and selected randomly from the selected districts of the Horo Guduru Wollega zone (Shambu, Hareto, Finchaa, and Sakela) were the study population.

### 2.4 Sample size determination

The sample size of the study was calculated using the formula for a single proportion of the population. The following assumptions were used during the sample size calculations; Z/2 = the critical value for the normal distribution at the 95% confidence level is 1.96 (z-score at alpha = 0.05), d is the margin of error (5%), p is the proportion of household food insecurity increased during Covid-19 in Ethiopia 12.13% [67]. Therefore, the total sample size was 361 participants, allowing for a design effect of 2 and a non-response rate of 10%.

### 2.5 Study variables

#### 2.5.1 Dependent variables

Household food security scores and dietary diversity were used as dependent variables in this study. In this, study, household food security is considered as a categorical variable (0 = food security and 1 = food insecurity). Household dietary diversity is used as continuous variable and expressed in mean and standard deviation. The Household Food Insecurity Access Scale (HFIAS) tool was used, which included nine of his questions about family experiences over the past month (last 30 days before the survey). Based the Food and Agricultural organization guidelines, households were classified as insecure, moderate, and secure households [68]. However, for this study two levels (food insecure and secure households) were used due to small sample size of the study [69].

A structured 24-h meal recall questionnaire was used to calculate the diversity score of household diets over the past 24-h. Respondents were asked to indicate the food they had eaten at home and outside, in the last 24-h. The food groups (11) used in this study were cereals, tubers, legumes, meat, eggs, vegetables, fruits, oils, sweets, milk and fish. Finally, household dietary diversity scores were assessed in mean and standard deviation over the previous 24 h [70,71].

#### 2.5.2 Independent variables

The independent variables included in the logistic regression analysis were socio-demographic characteristics such as monthly income, family size, educational status occupational status, unplanned child delivered after Covid-19 pandemic, food security, dietary diversity, knowledge, attitudes, and practices toward Covid-19. These variables were collected using structured questionnaires developed from previously published literatures [37,39,72].

### 2.6 Sampling procedures and data collection method

The Horo Guduru Wolega Zone was intentionally selected as a study site due to the lack of research data on household food security and dietary diversity after the Covid-19 pandemic and related factors. Therefore, we used a multilevel sampling method to extract a sample for investigation. First, the towns of Shambu, Hareto, Finchaa, and Sakela were randomly selected from a total of 14 towns in the Horo Guduru Wollega zone. Households in each town are then sized using the percentage of the population.

Finally, all participants (361) required for the study were randomly selected from each town. Data collection was conducted using structured personal questionnaires administered by interviewers, and after reviewing the relevant literatures, socio-demographic characteristics, household food security, dietary diversity, knowledge of coronavirus, attitudes, and practices were included. To ensure data quality, the questionnaire was written in English, translated into the local language (Afaan Oromoo), and translated into English by a bilingual expert fluent in both languages to ensure consistency. Two days training were given for data collectors before the actual data collection began. Pre-tests were conducted in nearby unselected towns. Data were collected by 12 enumerators, 4 supervisors, and his team co-investigators. At the end of each day, the completeness of the questionnaire was checked by the study administrator.

### 2.7 Statistical analyses

Data were analyzed with SPSS statistical software version 25.0. Descriptive statistics were used and presented using tables and percentages. Continuous variables are presented as mean and standard deviation, whereas categorical variables are presented as frequencies and percentages. The normality of continuous variables was checked using a one-sample Kolmogorov-Smirnov test. Bivariate and multivariate logistic regression models were used to identify factors associated with food security and dietary diversity. The suitability of the model was checked using the Hosmer and Lemeshow suitability tests. P values less than 0.05 were considered statistically significant. The degree of association between dependent and independent variables was expressed using an adjusted odds ratio with 95% CI.

### 2.8 Ethical approval and consent to participate

All procedures were approved by the Institutional Review Board (IRB) of the Wollega University Institute of Health Sciences after protocol review (reference RRC: no/WU/0246/2021). Local authorities were informed of the study by an official letter from the university. Respondents were fully and accurately informed about the study and their right to participate or discontinue at any time. Written informed consent was obtained from each participant and confidentiality was maintained throughout the study.

## 3 Results

### 3.1 Household characteristics

A total of 361 respondents (100% response rate) took part in this study. In addition, the mean and standard deviations of respondents' age and family size were 48.03 ± 7.659 and 5.48 ± 1.872, respectively. Sixty-eight of surveyed participants were delivered unplanned children after the Covid-19 pandemic. In addition, about 240 (66.5%) of the respondents had a monthly income of less than 10 USD and the detail information is presented in the table below (Table 1).

**Table 1:** Socioeconomic characteristics.

Variables	Frequency (n)	Percent (%)
Head of the household		
Mother	36	10.0
Father	325	90.0
Educational status		
Illiterate	94	26.0
Able to read and write	67	18.6
Elementary school and above	200	55.4
Marital status		
Married	325	90.0
Widowed	36	10.0
Religion		
Orthodox	93	25.8
Protestant	233	64.5
Muslim and other	35	9.7
Ethnicity		
Oromo	291	80.6
Amhara and other	70	19.4
Occupational status		
Household wife only	8	2.2
Unemployed	210	58.2
Labor	143	39.6
Unplanned child delivered after Covid-19 pandemic emerged		
Yes	68	18.8
No	293	81.2
Age of the respondent in mean and standard deviation	48.03 ± 7.659	
Family size in mean and standard deviation	5.48 ± 1.872	
Household income per month		
<10 USD	240	66.5
≥10 USD	121	33.5

### 3.2 Household food security

Seventy-eight (21.6%) of the participants were concerned that their households would not have enough food in the past month before the survey, and about 128 (35.5%) household members were unable to eat their favorite foods (Table 2). In addition, 215 (59.6%), and 146 (40.4%) of the respondents came from food-insecure, and secure households, respectively.

**Table 2:** Household food security status.

Variables	Frequency (n)	Percent (%)
Worried about household would not have enough food		
No	78	21.6
Rarely	128	35.5
Sometimes	123	34.1
Often	32	8.9
Household member not able to eat the kinds of foods you preferred		
No	128	35.5
Rarely	87	24.1
Sometimes	146	40.4
Household members have to eat a limited variety of foods due to a lack of resources		
No	107	29.6
Rarely	30	8.3
Sometimes	81	22.4
Often	143	39.6
Household members have to eat some foods that they did not want to eat		
No	242	67.0
Rarely	58	16.1
Sometimes	61	16.9
Household members have to eat a smaller meal than they felt you needed		
No	127	35.2
Rarely	57	15.8
Sometimes	161	44.6
Often	16	4.4
Household members have to eat fewer meals in a day		
No	157	43.5
Rarely	93	25.8
Sometimes	111	30.7
No food to eat of any kind in your household		
No	262	72.6
Rarely	38	10.5
Sometimes	61	16.9
Household members go to sleep at night hungry		
No	293	81.2
Rarely	68	18.8
Household members go a whole day and night without eating anything		
No	301	83.4
Rarely	60	16.6
Household food security Status		
Food Insecure	215	59.6
Food Secure	146	40.4

### 3.3 Household dietary diversity

Almost 336 (93.1%) and 281 (77.8%) of the respondents consumed cereals and legumes, respectively (Table 3). The consumption of meat (6.1%), eggs (11.9%), and fish and seafood (1.1%) was low. In addition, the mean and standard deviation of the values for dietary diversity scores was 4.19 ± 1.844 before the survey.

**Table 3:** Household dietary diversity.

Variables	Frequency (n)	Percent (%)
Cereals (teff, sorghum, maize, millet, wheat, barley)		
No	25	6.9
Yes	336	93.1
Pulses (beans, peas, chickpeas, lentils, nuts)		
No	80	22.2
Yes	281	77.8
Tubers (carrot, sweet potato, cassava)		
No	301	83.4
Yes	60	16.6
Vegetables (Lettuce, green cabbage)		
No	282	78.1
Yes	79	21.9
Fruits (Mango, papaya, banana, etc)		
No	321	88.9
Yes	40	11.1
Meat (Lamb, goat, beef, chicken)		
No	339	93.9
Yes	22	6.1
Eggs		
Yes	318	88.1
No	43	11.9
Fish and seafood		
No	357	98.9
Yes	4	1.1
Milk and milk products		
No	290	80.3
Yes	71	19.7
Oil and other butter		
No	65	18.0
Yes	296	82.0
Sweets (Sugar, bee honey)		
No	251	69.5
Yes	110	30.5
Household dietary diversity score	Mean (St. deviation)	4.19 ± 1.844

### 3.4 Knowledge of respondents towards Covid-19 pandemic

About 42.9% and 36.3% of respondents were unaware that Covid-19 is a viral infection transmitted through close contact with an infected person (Table 4). About 164 (45.7%) of surveyed participants indicated that children and adolescents were not at risk of death from Covid-19. Additionally, one hundred and thirty-five (37.4%) respondents had little knowledge of Covid-19 pandemic.

**Table 4:** Knowledge of respondents towards Covid-19 pandemic.

Variables	Frequency(n)	Percent (%)
Have you heard information about Covid-19?		
No	29	8.0
Yes	332	92.0
Is Covid-19 a virus infection?		
No	106	29.4
Yes	100	27.7
I don’t know	155	42.9
Is Covid-19 transmitted by close contact with the infected person?		
No	63	17.5
Yes	167	46.3
I don’t know	131	36.3
Are fever, fatigue, dry cough, and shortness of breath symptoms of Covid-19?		
No	204	56.5
I don’t know	157	43.5
If one gets a cold, cough, or fever he/she is Covid-19 infected		
No	100	27.7
Yes	261	72.3
The best way to prevent Covid-19 is to avoid the crowd and stay at home		
No	30	8.3
Yes	297	82.3
I don’t know	34	9.4
Children and young have no risk of death after Covid-19 pandemic		
No	100	27.7
Yes	165	45.7
I don’t know	96	26.6
If a crowd happens there is no chance of Covid-19 spreading		
No	164	45.4
Yes	68	18.8
I don’t know	129	35.7
Covid-19 can only go inside the body through the nose but not through the eyes and mouth		
No	134	37.1
Yes	227	62.9
A person who looks healthy and has no cough or fever can’t spread Covid-19		
No	61	16.9
Yes	264	73.1
I don’t know	36	10.0
Knowledge status of the respondents		
Poor	135	37.4
Good	226	62.6

### 3.5 Attitude of respondents toward Covid-19 pandemic

The result of this study shows that about 137 (38%) of the respondents believed that the black race protects against Covid-19 disease. One hundred and thirty-four (37.1%) respondents felt that using a hand wash would not protect them from contracting Covid-19 pandemic (Table 5). Approximately 160 (44.3%) participants had negative attitudes towards the Covid-19 pandemic.

**Table 5:** Attitude of respondents towards pandemic.

Variables	Frequency(n)	Percent (%)
Do you think the Covid-19 situation will stay under control?		
No	226	62.6
Yes	135	37.4
Do you think the black race is protected against Covid-19 disease?		
No	224	62.0
Yes	137	38.0
Do you think wearing a well-fitting face mask is effective in preventing Covid-19?		
No	167	46.3
Yes	194	53.7
Do you think using a hand wash can prevent you from getting Covid-19?		
No	134	37.1
Yes	227	62.9
Do you think eating pepper and garlic can prevent you from getting Covid-19?		
No	128	35.5
Yes	233	64.5
Attitude status of the respondents		
Negative	160	44.3
Positive	201	55.7

### 3.6 Practices of respondents towards Covid-19 pandemic

More than half (52.9%) of respondents do not wash their hands with soap or other antiviral materials after returning home. Almost 73.4% of respondents did not wear masks when going out (Table 6). Additionally, 153 (42.4%) of respondents had bad practices during the Covid-19 pandemic.

**Table 6:** Practices of respondents towards Covid-19 pandemic.

Variables	Frequency(n)	Percent (%)
Do you stay your home during the Covid-19		
No	325	90.0
Yes	36	10.0
Do you wash your hands with soap or other anti-viral materials when returning home?		
No	191	52.9
Yes	170	47.1
Do you wear masks when going outside the home?		
No	265	73.4
Yes	96	26.6
Do you maintain a safe distance from people (more than 2 m) when going outside the home?		
No	227	62.9
Yes	134	37.1
In recent days, have you gone to any crowded places?		
No	95	26.3
Yes	266	73.7
In recent days, have you refrained from shaking hands?		
No	153	42.4
Yes	206	57.1
Practices of the respondents		
Poor	153	42.4
Good	208	57.6

### 3.7 Binary and multivariable logistic regression analysis

#### 3.7.1 Factors associated with household food security

Multivariate analysis of this study showed that family size, monthly income in $USD, dietary diversity, knowledge, attitude and practices towards the coronavirus were factors associated with food insecurity (Table 7).

**Table 7:** Binary and multivariable logistic regression of factors associated with household food security after the Covid-19 pandemic emerged in Ethiopia.

Variables	COR (95% CI)	AOR (95% CI)
Educational status		
Illiterate	1.27 (0.658, 2.452)	1.354 (0.391, 4.689)
Able to read and write	1.78 (1.064, 2.98)	1.36 (0.469, 3.954)
Elementary school and above	1	1
Occupational status		
Household wife only	1	1
Unemployed	5.4 (1.053, 27.8)*	0.181 (0.017, 1.922)
Labor and other	1.33 (0.856, 2.056)	0.273 (0.111, 0.667)
Family size increased after the Covid-19 pandemic		
Yes	2.86 (1.54, 5.299)*	1.412 (0.544, 3.68)
No	1	1
Family size		
<5	1	1
≥5	1.36 (0.86, 2.159)	8.5 (3.295, 21.92)**
Household monthly income		
<10USD	3.88 (2.45, 6.147)*	3.52 (1.771, 6.986)**
≥10USD	1	1
Dietary diversity scores		
Less than mean	7.022 (4.357, 11.32)*	8.5 (3.92, 18.59)**
Greater than or equal to mean	1	1
Knowledge		
Poor	5.7 (3.6, 9.138)*	3.0 (1.08, 8.347)**
Good	1	1
Attitude		
Negative	7.64 (4.604, 12.66)*	8.35 (3.112, 22.39)**
Positive	1	1
Practices		
Poor	2.83 (1.14, 16.947)*	2.12 (1.299, 11.4)**
Good	1	1

AOR; adjusted odds ratio, COR; Crude odds ratio, *; Significant variables in bivariate analysis (p < 0.25),**; significant variables in multivariate (p < 0.05) analysis 1; reference.

#### 3.7.2 Factors associated with dietary diversity

This study result also indicates that educational status, unplanned child delivered after Covid-19 pandemic emerged, household food security, knowledge, attitude and practices were predictors significantly associated with dietary diversity (Table 8).

**Table 8:** Binary and multivariable logistic regression of factors associated with household dietary diversity after the Covid-19 pandemic emerged in Ethiopia.

Variables	COR (95% CI)	AOR (95% CI)
Educational status		
Illiterate	3.8 (1.739, 8.362)*	2.558 (0.944, 6.93)
Able to read and write	6.31 (3.24, 12.28)*	3.46 (1.44, 8.312)**
Elementary school and above	1	1
Occupational status		
Household wife only	1	1
Unemployed	5.4 (1.053, 27.8)*	4.03 (0.43, 37.66)
Labor and other	1.002 (0.643, 1.562)	1.338 (0.686, 2.61)
Family size increased after Covid-19 pandemic		
Yes	3.24 (1.664, 6.298)*	2.26 (1.02, 5.04)**
No	1	1
Family size		
<5	1	1
≥5	1.442 (0.913, 2.276)	0.978 (0.442, 2.163)
Household monthly income		
<10 USD	0.699 (0.446, 1.096)	1.349 (0.745, 2.442)
≥10 USD	1	1
Household food security status		
Food insecurity	7.022 (4.357, 11.32)*	6.7 (4.01, 19.01)**
Food security	1	1
Knowledge		
Poor	4.13 (2.62, 6.515)*	3.96 (1.57, 10.0)**
Good	1	1
Attitude		
Negative	4.46 (2.75, 7.24)*	3.9 (1.75, 8.82)**
Positive	1	1
Practices		
Poor	3.95 (1.85, 4.69)*	2.23 (2.18, 23.95)**
Good	1	1

AOR; adjusted odds ratio, COR; Crude odds ratio, *; Significant variables in bivariate analysis (p < 0.25),**; significant variables in multivariate (p < 0.05) analysis 1; reference.

## 4 Discussion

The prevalence of food insecurity (59.6%) in the current study is lower than in previous studies conducted in Bangladesh [33], Indonesia [73], and Kenya [74], which reported 93.2%, 65.0%, and 91.0% of food insecurity after the Covid-19 pandemic emerged in a region. However, it is higher than a study done in Ethiopian (6.8%) and Bangladesh (7.18%) of urban households [23]. The study results are also higher than those of a previous study conducted in the United States, which found that about 14.7% of the participants had low household food security, with a higher prevalence (17.5%) in households with children [75]. This disparity could be attributed to socioeconomic factors, sample size, and data collection period.

The mean and standard deviation of the current dietary diversity score (4.19 ± 1.844) is higher than that of a study from Bangladesh, which found that the average total household dietary diversity score was 4.08 ± 15 [31]. However, it is lower than the results of previous studies in Bangladesh [34], and Kenya [74], which reported mean scores for household dietary diversity of 8.6 ± 1.5 and 6.22 ± 5.49, respectively. The inequality could be explained by the fact that people’s dietary habits differ from region to region.

According to the results of this study, households with a family size≥5 were 8.5 times more likely to be food insecure than households with fewer than five children. This finding is consistent with previous studies in Bangladesh [[31], [32], [33], [34]]. This could be because households with large families are more likely to be food insecure after the Covid-19 pandemic emerged in a country and unable to adequately feed their families due to loss of employment and lack of work.

Households with a monthly income of less than 10 USD were 3.52 times more likely to be food insecure than households with ≥10 USD before the survey. This result is consistent with studies conducted in Ethiopia [23], Bangladesh [[31], [32], [33]], and Indonesia [73]. This could be because household income plays a role in encouraging households to provide enough food, which can improve food security.

The multivariate logistic regression analysis of the current study shows that households with less than mean values for dietary diversity were 8.5 times more likely to be diet-insecure households than households with greater than or equal to mean values for dietary diversity scores. The result of this study is consistent with other studies conducted in Iran [30], Bangladesh [32], Jordan [75], Kenya and Uganda [76], and Peru [77]. This could be because low dietary intake is common among poor urban communities, and it can cause households to become insecure as they are highly interconnected.

The results of this study shows that after the Covid-19 emerged in a country, illiterate people are 3.46 times more likely to have less than mean values for dietary diversity than those who were in elementary school or higher. The result of this study is consistent with other studies conducted in Bangladesh [34], Ethiopia [78], and Iran [79]. This could be due to knowledge and information gaps regarding the practices of Covid-19 transmission between illiterate and educated people. In terms of family size, households that gave unplanned birth and enlarged families after the Covid-19 pandemic in a nation were 2.26 times more likely to have less than mean values for dietary diversity than those that did not. This may be because when the Covid-19 pandemic emerges in a country, it may limit households' capacity to purchase and consume various foods from the market.

Another variable significantly associated with less than mean values for dietary diversity was food security. Food-insecure households were 6.7 times more likely to have poor food intake than their peers. This study finding is consistent with studies conducted in Saudi Arabia, Iran, Bangladesh and Jordan; which reported that there was a significant association between food insecurity and reduced dietary values during the Covid-19 pandemic [28,30,34,75]. This could be explained by the fact that food shortages after the Covid-19 pandemic emerged may have affected household food consumption and caused households to consume less varied foods.

According to the results of this study, 37.4%, 42.4% and 44.3% of the respondents had poor knowledge, negative attitudes, and bad practices related to the Covid-19 pandemic, respectively. The results of this study are similar to those of an Ethiopian systematic review, which found that the overall estimated status of poor knowledge, negative attitude, and bad practices on the coronavirus in Ethiopia is 38.2%, 27.6%, and 47.2%, respectively [52]. However, the current study results are lower than those of an Indonesian study that revealed poor knowledge (74.7%), negative attitudes (63.4%), and poor practices (51.2%) toward Covid-19 [80]. The negative attitude of the current study is also lower than the negative attitude (53.7%) towards the Covid-19 pandemic in northeastern Ethiopia [66]. This could be because the level of knowledge, attitudes, and practice towards the coronavirus depends on information, the level of education of the study participants, resources, and the ability to use technology.

The current poor knowledge, attitudes and practices of the study results is higher than a study conducted in Ethiopia, which found that the overall estimated poor knowledge, negative attitude, and poor practice towards Covid-19 were 20.6%, 26.3%, and 40.3%, respectively [81]. However, the current study results are lower than those of an Indonesian study that revealed low knowledge (74.7%), negative attitudes (63.4%), and bad practices (51.2%) toward Covid-19 [80]. The negative attitude of the current studies is also lower than the negative attitude (53.7%) towards the Covid-19 pandemic in northeastern Ethiopia [82]. This could be because the level of knowledge, attitudes, and practice towards the coronavirus depends on information, the level of education of the study participants, resources, and the ability to use technology.

During the survey, respondents who had poor knowledge, negative attitudes, and poor practices toward Covid-19 were 3.0, 8.35, and 2.12 times more likely to be in diet-insecure households, respectively (Table 7). Respondents with poor knowledge, negative attitudes, and bad practices about Covid-19 were also 3.96, 3.9, and 2.23 times more likely to have low diet variety than their peers, respectively (Table 8). A similar finding from Lebanon showed that attitudes toward dietary diversity values changed when the pandemic struck [83]. It also agrees with another study result from Iran [30]. A qualitative study conducted in Ethiopia also reported that knowledge, attitudes, and practices were not sufficient to combat and minimize the impact of the Covid-19 pandemic [84]. This could be because people with poor knowledge, perception, and practices may not understand information about the problems and are unable to ensure household food security and dietary diversity.

## 5 Limitations and future research directions

We acknowledge a couple of limitations in our study. Our study findings are limited by the small sample size of population, the study was conducted over a single period, so the results may not accurately reflect household food security and dietary diversity. In addition, this study was a cross-sectional, so no cause-and-effect relationship could be established between the predictors and the outcome variables. Moreover, the results of this study may have been influenced by memory biases during the interview. This study was conducted in urban areas and may not tell us what factors are really associated with food security and dietary diversity in rural settings. Lack of literatures and published articles in a region regarding this topic is also a big problem to compare the household food insecurity and dietary diversity results with other regions of Ethiopia. Conflicts and political instability during the survey in a region may also overestimate the prevalence’s of household food insecurity and dietary diversity. As a result, future researchers are advised to conduct this research in a different context, which may include other types of districts, regions and cultures. Furthermore, the various rural areas of Ethiopia should not be overlooked, as this study only focused on the urban areas. As a result, it is critical to examine respondents from various geographical locations.

## 6 Strengthens of the study

This study was a community based cross-sectional and estimated the prevalence of food insecurity among poor urban communities after the outbreak of Covid-19 pandemic. The mean and standard deviation of household dietary diversity was also calculated. The study was conducted during a serious situation in which data collection and gathering information from participants is very difficult. The study used standard questionnaires and logistic regression analyses to identify the main factors associated with food security and dietary diversity among poor urban communities after the Covid-19 pandemic in a region.

## 7 Conclusion

The Covid-19 pandemic impacted the reduction in household food security scores and dietary diversity among urban residents of the nation and study area. This study examined in detail the main factors significantly associated with household food security and diet intake in poor urban households after the outbreak of the Covid-19 pandemic. The prevalence of food household insecurity and less mean values for dietary intake is higher in the current study than in the region and nation. This may require cross-sectorial collaboration that can help vulnerable communities obtain adequate food through social safety nets, with the goal of ensuring food security and further improving food security. The levels of poor knowledge, negative attitudes, and poor practices among study participants were rated as low compared to other studies conducted in the region. Therefore, raising awareness of the Covid-19 pandemic in urban communities is crucial to prevent transmission of the infection and reduce healthcare costs. In the multi-logistic regression analysis of this study, the variables that were significantly related to household food security were family size, monthly income, dietary diversity, knowledge, attitudes, and practices after outbreak of the Covid-19 pandemic. The study’s finding also shows that educational status, unplanned child delivered after the Covid-19 pandemic, household food security status, knowledge, attitude, and practices were important predictors of household dietary diversity score. This study can also help inform governments to use nutrition-specific and nutrition-sensitive programs where their efforts need to be focused to reduce food insecurity and improve the health and nutritional status of vulnerable communities. Therefore, the Ethiopian Ministry of Health should strengthen health education and promotion through mass media to improve communities' knowledge, attitude and practices toward coronavirus. In addition, health extension workers should encourage communities to participate in social work to get money and secure their food consumption problems. Furthermore, agricultural agent developments should help communities to participate on income generating activities like urban home gardening and small animal breeding to ensure their variety of foods and to generate income. Food and nutrition policies and strategies in a country should be strengthened by all responsible agencies to address household-level factors affecting food security and nutritional intake of poor urban communities.

## Declarations

### Author contribution statement

Tamiru Yazew: Conceived and designed the experiments; Performed the experiments; Analyzed and interpreted the data; Contributed reagents, materials, analysis tools or data; Wrote the paper.

Agama Daba, Lelisa Hordofa, Girma Garedew, Abdi Negash, Gizachew Merga, Tasama Bakala: Performed the experiments; Analyzed and interpreted the data; Wrote the paper.

### Funding statement

This research did not receive any specific grant from funding agencies in the public, commercial, or not-for-profit sectors.

### Data availability statement

Data will be made available on request.

### Declaration of interest’s statement

The authors declare no conflict of interest.
